# Supplementary material for: BlueFeather, the singleton that wasn’t: Shared gene content analysis supports expansion of Arthrobacter phage Cluster FE
Source: PLoS One. 2021 Mar 12;16(3):e0248418. doi: 10.1371/journal.pone.0248418 (PMC7954295; doi:10.1371/journal.pone.0248418)
Supplement: Attachment — Submitted filename: Bluefeather Manuscript Response to Reviewers.docx [file pone.0248418.s001.docx]

**1/20/2021**

**Bluefeather Manuscript Response to Reviewers**

Dear PLOS ONE Editor,

Thank you for the opportunity to revise and resubmit our manuscript. We have addressed the editor and reviewer comments below.

Journal Requirements:

1. The “BlueFeather Revised Manuscript Reformatted_01-20-2021” document has been reformatted according to the PLOS ONE style template.
2. Reference to funding removed from Acknowledgements section, and it has been revised to thank the department and division for general programmatic support. The original financial disclosure is correct: “The authors received no specific funding for this work.”

Response to Reviewer 1:

Thank you for the positive feedback regarding the writing, data, and analyses. We respectfully disagree with the reviewer’s suggestion that the conclusion is not scientifically novel or important enough for publication in PLOS ONE. In addition to reclassifying the novel phage BlueFeather from singleton to Cluster FE, this work did also result in the re-organization of the previously distinct Cluster FI into an expanded Cluster FE. Prior to this work, *Arthrobacter* phages were clustered using nucleotide identity as described by Klyczek *et al.* (2017), but now the Hatfull lab (which runs phagedb.org) has adopted the parameters we describe for clustering new *Arthrobacter* phages. We believe our paper has the strong scientific validity, methodology, and ethical standards necessary for publication in PLOS ONE, which doesn’t select articles based on perceived significance.

Response to Reviewer 2 (Minor Issues to Address):
• Figure 1A: The plaques are difficult to see and are described as being 5mm in diameter though there is a mixture in plaques sizes on this plate. On pg 7, line 153-155, the figure is described as a plate produced from an infection using a picked plaque, so is this from purification steps before the high titer lysate was produced? Perhaps a clearer image of plaques from the lysate would be better and the plaques would be consistent in size. If plaques are variable in size for this phage, that is not uncommon and should be stated.

**Thank you for the suggestion, we went back and used ImageJ to calculate average plaque diameter (sentence added to methods at lines 99-100). A revised figure 1A has been submitted with an inset showing a magnification of the plaques, and a revised figure legend. The results section has been revised to reflect a more detailed description of plaque size and morphology variations (lines 150-153).**

• Pg 9, line 219: In the text, Idaho has 34.18% shared gene content with BlueFeather while in Figure 4A itself it says that Idaho has 38.5% shared gene content with BlueFeather (though the text in the figure is hard to read in the pdf). These numbers should be consistent. 38.5% GCS would provide an even stronger argument for clustering BlueFeather with Idaho.

**The GCS calculation was repeated confirming the 38.5% GCS for Phage Idaho (text updated in lines 221-223). The repeated analysis also indicated that the MaxGCDGap was between BlueFeather and Yavru (41.60%), rather than Whytu. The text (line 230), Figure 4b and legend have been revised.**

• Pg 5, line 96: A reference should be provided for phage purifications or details about the procedure should be included.

**Reference added at line 99.**

• Pg 16, line 373: may I suggest changing this sentence so that it flows better to ‘BlueFeather serves as yet another example of the highly intricate mosaic relationships which exist among phages and are a feature of the genetic landscape, making phage taxonomy an increasingly difficult task.’

**Thank you for the suggestion, the sentence has been edited.**

**The following suggested edits have been incorporated:**
• Pg 3, line 62: comma needed after phylogeny
• Pg 5, line 103: comma after (Philips, Amsterdam, Netherlands)

• Pg 6, line 126: may I suggest changing ‘whole amino acid sequences’ to ‘whole proteome amino acid sequences’ to clarify that the entire proteome was examined

**The suggested edits below change the meaning of the text, so the sentences were changed from “which exhibited” to “exhibiting” to improve clarity (lines 232 and 240).**

• Pg 10, line 228: comma between phages and which
• Pg 10, line 229: comma between 1 and were
• Pg 10, line 235: comma between phages and which
• Pg 10, line 236: comma between 1 and were

We would like to thank Reviewer 2 for the detailed feedback, which helped improve the quality of our manuscript.

Sincerely,

Jordan Moberg Parker, Ph.D.
